# Adapting to Aging: Older People Talk About Their Use of Selection, Optimization, and Compensation to Maximize Well-being in the Context of Physical Decline

**DOI:** 10.1093/geronb/gbw132

**Published:** 2016-10-21

**Authors:** J. D. Carpentieri, Jane Elliott, Caroline E. Brett, Ian J. Deary

**Affiliations:** 1 UCL Institute of Education, London, UK.; 2 Liverpool John Moores University, UK.; 3 MRC-BBSRC Centre for Cognitive Ageing and Cognitive Epidemiology, Department of Psychology, University of Edinburgh, UK.

**Keywords:** Adaptation, Narrative, SOC, Successful aging, Well-being

## Abstract

**Objectives:**

Selection, Optimization, and Compensation (SOC) may contribute to successful aging by helping older people maximize well-being in the context of physical decline. To explore this hypothesis, and to investigate the potential for narrative analysis to improve understanding of SOC, we analyze interviews conducted with 15 members of the 6-Day Sample, a cohort of Scots born in 1936.

**Method:**

Interviewees were chosen based on their physical function and well-being scores. Interviews were analyzed to investigate “SOC talk,” that is, older people’s talk about SOC behaviors in everyday life. Types and amounts of SOC talk were quantified, and talk was narratively analyzed. We hypothesized that older people who engaged in more SOC talk would have higher well-being.

**Results:**

Older people who engaged in high levels of SOC talk had high well-being despite low physical function. Those who engaged in little SOC talk had low well-being despite higher physical function.

**Discussion:**

The concept of successful aging is valuable in part because of its narrative quality: One must strive to keep one’s life story developing despite physical decline and other losses. We provide evidence, from the perspectives of older people themselves, of the ways in which SOC may play a role in that process.

The concept of successful aging has prompted debates over how to define success and who gets to define it (Bülow & Söderqvist, 2014; Katz & Calasanti, 2015; Martin et al., 2015). Most models of successful aging emphasize physical function, often in combination with other outcomes (Depp & Jeste, 2006; Rowe & Kahn, 1997, 1998). Other models (e.g., Hsu & Jones, 2012) stress the importance of subjective well-being. Although physical health and subjective well-being are typically highly correlated, there are significant numbers of older people with “off-diagonal” profiles, for example, high well-being despite low physical function (Zammit, Starr, Johnson, & Deary, 2012, 2014). Such profiles give rise to a central question in the study of aging, particularly as average life spans increase (Cho, Martin, & Poon, 2012): How can older people achieve high well-being despite the physical decline that is characteristic of most people’s experience of aging?

One potential mechanism is Selection, Optimization, and Compensation (SOC; Baltes & Baltes, 1990), a model of successful aging that focuses not on outcomes but on the processes individuals engage in to maximize gains and minimize losses in response to everyday demands and functional decline in later life (Baltes & Carstensen, 1996). Like other process models, the SOC framework offers a conceptualization of success that is not outcome dependent, but centers on doing the best one can with what one has (Strawbridge, Wallhagen, & Cohen, 2002). As originally conceptualized, Selection focused on goal choice, while Optimization and Compensation focused on the means for achieving goals (Baltes & Baltes, 1990; Baltes & Carstensen, 1996). Some researchers (e.g., Gignac, Cott, & Badley, 2002; Janke, Son, & Payne, 2009; Lang, Rieckmann, & Baltes, 2002; Rozario, Kidahashi, & DeRienzis, 2011) have extended the conceptualization of SOC to focus on the *activities* that individuals engage in as they pursue their goals. Such activities represent “goal-directed instrumental behaviors” (Kahana, Kahana, & Lee, 2014, p. 470) and may represent “selection, optimization, and compensation in everyday functioning” (Lang et al., 2002, p. 507). This extended, activity-focused conceptualization of SOC is in line with other process models, for example, the Preventive Corrective Proactive model (Kahana et al., 2014, p. 468), which includes a focus on “specific behavioral actions undertaken by older individuals to actively deal with impending or extant aging-related stressors.”

SOC and other adaptive processes have been found to be positively associated with indicators of successful aging, such as well-being, life satisfaction, and quality of life (Freund, 2008; Freund & Baltes, 1998; Jopp & Smith, 2006; Kahana, Kelley-Moore, & Kahana, 2012; Ouwehand, de Ridder, & Bensing, 2007), and may be particularly helpful for low-resource individuals in their efforts to maximize well-being (Freund, 2008). These findings suggest that whereas the likelihood of successful aging is positively associated with physical function, successful aging may coexist with poor physical health if adaptive strategies are used (Young, Frick, & Phelan, 2009). Kahana and colleagues (2014, p. 468) argue that whereas some older people are “lucky agers,” who suffer minimal physical decline, most older people must adapt to physical loss in order to maintain well-being. Process models can provide insights into behaviors that enhance this process.

Rowe and Kahn (2015) have recently argued that process models should play a more central role in successful aging research. They have also supported calls for researchers to address the “missing voices” critique (Martinson & Berridge, 2015) by paying more attention to older people’s own perspectives on aging. Though research on lay perspectives on aging (Bowling & Dieppe, 2005; Cosco, Prina, Perales, Stephan, & Brayne, 2013; Jopp et al., 2015) has helped address this critique, the field of successful aging in general and SOC in particular is characterized by an “etic” (outsider) approach (Guba & Lincoln, 1994), which provides helpful evidence on propensities towards SOC, but sheds less light on how and why older people incorporate SOC into their daily lives. Exceptions include the content analysis approach of Gignac and colleagues (2002), who quantify amounts and types of talk about SOC, and Rozario and colleagues’ (2011) thematically focused approach, which explores SOC’s influence on self-concept.

In this article, we bring together and build on these approaches. We do so using two main analytic approaches. Using content analysis (Franzosi, 2008), we identify and enumerate “SOC talk,” that is, older people’s talk about SOC use in their daily lives. Our coding and quantification of SOC talk allows investigation of the hypothesis that older people who maintain high well-being despite low physical function will report greater use of SOC than their “on-diagonal” peers and that individuals who report low well-being despite relatively high physical function will report less use of SOC. Next, we undertake narrative analysis (Riessman, 1993) of SOC talk. This approach allows exploration of the ways in which SOC may help older people pursue goals and maximize well-being. In other words, we do not simply identify and quantify talk about SOC; we also analyze how older people embed this talk in accounts and anecdotes of daily life. We focus in particular on SOC talk in narratives of physical function and activity. In doing so, we explore SOC through the lens of narrative gerontology (Kenyon, Clark, & de Vries, 2001), which views the self in terms of a story that one seeks to continually develop. Narrative has been argued to be a mechanism through which individuals can understand themselves as having a sense of self that endures over time without being fixed and unchangeable (Ricoeur, 1991). This interweaving of constancy and change may have particular salience in the study of aging, as individuals strive to maintain a “good strong story” in the context of physical or other losses (Carpentieri & Elliott, 2014). Narrative gerontology emphasizes the importance to well-being of avoiding narrative foreclosure (Freeman, 2000), that is, the sense that one’s life story has come to a conclusion, that there are no further opportunities for individual development and growth, and that one has therefore moved into “epilogue time” (Morson, 1994). In narrative terms, successful aging may thus be seen as involving the continued pursuit of goals, and the ongoing development or unfolding of one’s life story, even in the presence of physical decline and other losses (Andrews, 2009).

Whereas many studies of successful aging draw on samples from a broad age range, we focus on a purposively selected subsample of 15 Scots born in 1936. Our use of this cohort allows us to draw on quantitative data collected as part of a larger cohort study and to investigate the role of SOC in successful aging at a particular chronological age, the late 70s—a period when many adults may be on the borderline between an active Third Age (Laslett, 1989) and a Fourth Age characterized by physical decline and its impacts on well-being. In doing so, we adopt an “emic” or insider approach to SOC and seek to provide insights into the diverse ways that older people incorporate SOC into their lives and how this supports well-being, from the perspective of older people themselves.

## Method

**Figure 1.**
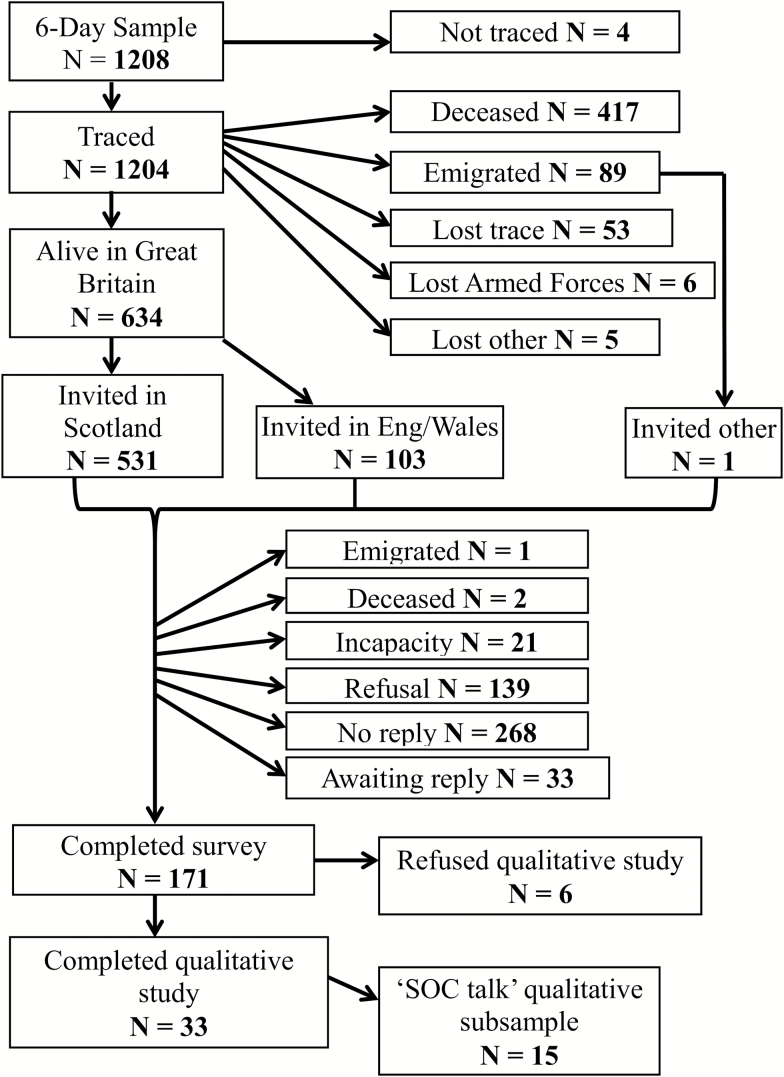
The 6-Day Sample study participants.

The 6-Day Sample of the Scottish Mental Survey 1947 follows a cohort of Scottish individuals born in 1936 (Deary, Whalley, & Starr, 2009; Scottish Council for Research in Education, 1949). Of the 1208 original members of the 6-Day Sample, later-life quantitative data, including well-being and physical function measures, were collected from 171 individuals at the age of 77 (Deary & Brett, 2015; see Figure 1). In the present article, well-being is represented by the Warwick-Edinburgh Mental Wellbeing Scale (WEMWBS; Tennant et al., 2007), which captures a broad conception of well-being, including positive affect, psychological functioning, and interpersonal relationships. Physical function is represented by the 10-question physical function subscale in the Medical Outcomes Study Short Form Health Survey (SF-36; Ware & Sherbourne, 1992). This captures self-reported ability to perform a range of activities, ranging from basic (e.g., dressing) to vigorous (e.g., running).

Table 1 provides current demographic information for the surviving sample. Sample attrition was somewhat higher for individuals with less education and lower occupational status (Johnson, Brett, Calvin, & Deary, 2016).

**Table 1.** Demographic Information of the Current Members of the 6-Day Sample Study

		Full sample, %	Qualitative subsample, %
		(*n* = 171)	(*n* = 33)
Gender	Female	53	52
	Male	47	48
Marital status	Married or civil partner	71	77
	Widowed	21	15
	Other	8	8
Housing tenure	Homeowner (with or without mortgage)	88	94
	Other	12	6

Following the collection of quantitative data, semistructured biographical interviews were conducted with a purposively selected subsample of 33 cohort members at the age of 77–78. The qualitative sampling framework took account of cohort members’ physical health, cognitive function, and psychological well-being (Carpentieri, Elliott, Brett, & Deary, 2016). Qualitative interviews were conducted by two experienced researchers. Questions covered a range of topics, including health, family, physical capabilities, interests, and activities, and included some questions that asked individuals to describe and discuss aspects of their lives, and others that were more evaluative (Table 2).

**Table 2.** Sample Qualitative Interview Questions

Question type	Question
Evaluative	Compared with someone about the same age as you, how would you say your health is? What goes through your mind when you say that?
	Does your health make it difficult to do anything that you used to do, for example, when you first retired?
	Are there any advantages of being your age? Are there any disadvantages?
	Some people say that older age is a time of loss. Others say it is a time of gain. What do you think?
Descriptive/activity focused	Could you talk me through your last week (including the weekend) in terms of how you spent your time?
	Do you do any regular physical activity or exercise, for example, walking, gardening, cleaning house or keep fit?
	Who does the cleaning, gardening, and maintenance? Do you get any help?
	Have you made any changes or adaptations to your home?

Interviews included no explicit questions about SOC. Open-ended questions were used to elicit narratives and concrete descriptions of individuals’ lived experiences (Chase, 1995; Holloway & Jefferson, 2000). The aim was for individuals to view the interviews as descriptions of their lives, rather than discussions of aging and/or adaptation per se. Thus, our questioning resonated with lay approaches to understanding aging, by allowing older people to focus on the aspects of life, which they found most meaningful. The biographical interviews were not seen as providing facts about respondents’ lives, but rather interviewees’ interpretations and representations of their lives (Kvale & Brinkmann, 2009). Transcripts were anonymized, handcoded to identify themes, and then coded by Carpentieri and Elliott using Nvivo10, allowing not only for detailed analysis of individual cases but also for comparative thematic analysis across groups of cases (Richards, 2014).

To investigate the role of SOC in potentially mediating the relationship between physical function and well-being, we first calculated residuals for all 171 study members by regressing well-being on physical function. Based on cohort members’ physical function and well-being, we then chose four conceptually distinct groups for our analysis of SOC talk. Group 1 had high well-being and physical function (top quartile in both measures). Group 2 scored in the bottom quartile in both measures. The other two groups were off-diagonal, in that their well-being scores were at least 1 *SD* higher or lower than would be expected given their physical function scores. Group 3 had high well-being despite low physical function, whereas Group 4 had low well-being despite relatively high physical function.

**Figure 2.**
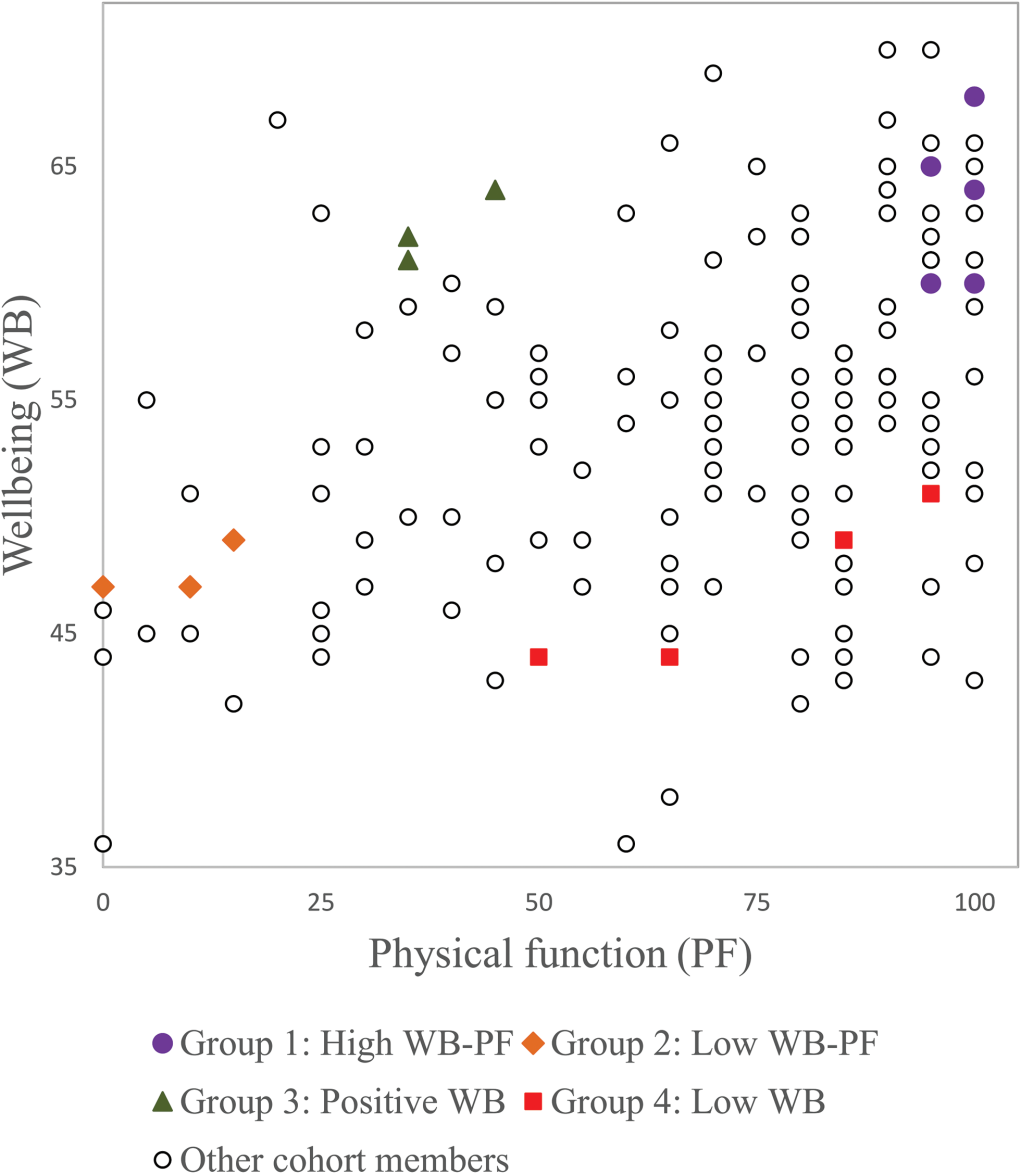
Physical function versus well-being, by Selection, Optimization, and Compensation talk groups.

Figure 2 illustrates the relationship between well-being and physical function in the full sample and highlights the 15 individuals and 4 groups included in our investigation.

### Coding and Analysis

Following the selection of our analytic subsample, we reanalyzed their interviews, counting and coding each instance of SOC talk related to physical function, including talk about physical activities. Our coding builds on earlier conceptualizations of activity-focused SOC in everyday life (Gignac et al., 2002; Lang et al., 2002). In such approaches, activities are seen as behavioral operationalizations of goal orientation. Whereas Lang and colleagues and Gignac and colleagues limited their coding of Selection to instances in which individuals restricted their activities, we suggest that such an approach does not take sufficient account of the potential for physical gains in older age, alongside losses. In our interviews, several individuals noted health improvements in their late 70s and described new activities they were engaging in and new goals they were pursuing as a result of these physical gains. We therefore classified SOC talk as Elective Selection (ES) if it involved activity selection that was not motivated by a decline in physical resources (Freund & Baltes, 1998, 2002). Loss-Based Selection (LBS) refers to activity choice in response to resource loss and implies a restriction in the range or intensity of activities (Gignac et al., 2002; Lang et al., 2002). Optimization refers to efforts to augment or enrich one’s capacities in order to continue functioning (Gignac et al., 2002). Compensation is the use of alternative means to pursue goals or maintain desired states in the context of resource loss, for example, by modifying behaviors or using assistive devices.

In our coding, we adopt a moderately broad measure of physical activity. In addition to more strenuous physical activities, we include those such as gardening, household repairs, and housekeeping; we do not include more sedentary activities such as knitting and driving. We include SOC talk about the individual him/herself, not talk about others. When narratives contained multiple examples of SOC, for example LBS and Compensation in the same narrative, or two examples of Compensation, each example of SOC was coded individually. Table 3 provides examples of SOC talk from the interviews. All names are pseudonyms, and identifying details have been removed.

**Table 3.** Selection, Optimization, and Compensation Talk Examples

Type of talk	Example
Elective Selection (ES)	Colin (Group 1) befriends someone who loves hillwalking, and he begins doing it as well. “Last Sunday…we walked to the top of Arthur’s Seat.”
	Brenda (Group 1) has recently started helping in a hospital garden. Recently, she said, “I have started helping in…a garden that’s been formed…it’s for staff and patients if they want come down and just walk around…. So I was along there, just doing what needs to be done.”
Loss-Based Selection (LBS)	Catherine (Group 3) said that she and her husband used to be “great hill walkers, but…that’s all by the board now because he can’t do it and I can’t do it. So we’ll walk to the hills rather than up the hills.”
	Ian (Group 4) has given up most physical activities, but continues to work in his garden, where, he notes, “The railway sleepers are rotten. They need to be replaced, so we’ve got the sleepers ready to go in and we’ll do that throughout the winter…. I’ll dig the holes and get on that, oh aye. You can’t just sit back and—, well, while you can do it, why not, you know? Sometimes I get a bit short of puff but, you know, when you get to my age it’s kind of acceptable with this sort of thing.”
Optimization (O)	Colin (Group 1) notes that he has been *“*making myself go to the gymnasium every day.” He does so in an effort to optimize his cognitive function: “My mental faculties were being dulled by the fact that I wasn’t physically active. Because I’ve now become more physically active I’m starting to regenerate the old thought processes. So it’s the interconnection between the physical ability to keep going, and the consequential result on the mental attitudes, which are very important.”
	Mary (Group 3) has suffered a number of falls in her large, sloping garden, and describes her efforts to avoid such falls by improving her physical stability: “My balance isn’t as good as it was. So I’m going to the next balance clinic next week.”
Compensation (C)	Eleanor (Group 2) said that because of her severe arthritis she sometimes struggles to clean her home, but manages, “with a little help from my friends, because I can’t get up high now, you know [and] I have long poles and things that help.” In this brief passage, Eleanor highlights two types of Compensation: help from friends and the use of assistive devices.
	Whereas Optimization focuses on increasing resources, Compensation focuses on strategies for making due with reduced resources. One such strategy is pacing, as Neil (Group 1) describes: “I think getting older makes—, you need to change your routine, you know. I’m looking at that hedge, and up until two years ago I would have cut this side, the top and the other side in the one go, but in the past two years I’ve left the other side for another day…. I just pace it. There was a question in [the 6-Day Sample quantitative] questionnaire that asked, have you ever had to stop doing a job in the middle of it, and I answered no, but I pace myself.”

## Results

### Amount and Types of Adaptation Talk

Table 4 shows the incidence of SOC talk engaged in by each group. The average number of SOC talk instances per individual was 12; this amount was not associated with interview length. The average incidence of SOC talk differed across groups but was consistent within groups: In three of four groups, no individual score was more than three points from the group average. The exception was Group 3. Individual totals and group averages provide support for our hypothesis that older people who maintained high well-being despite low physical function would report high use of SOC and that those with lower than expected well-being would report low use. All members of Group 3 (higher than expected well-being) engaged in above average amounts of SOC talk, with two members of this group having the highest totals in the sample. Group 4 (who had lower than expected well-being) accounted for the four lowest SOC talk totals in the sample. This evidence, while exploratory and based on a small sample, supports the hypothesis that SOC may mediate the relationship between physical function and well-being.

**Table 4.** Selection, Optimization, and Compensation Talk Points, by Group and Type of Talk

		Group 1: High WB–PF	Group 2: Low WB–PF	Group 3: Positive WB	Group 4: Low WB
Cohort members in group		5	3	3	4
Mean interview length, min		106	106	137	128
SOC instances per individual (mean)		12, 14, 14, 15, 16 (14)	12, 13, 13 (13)	14, 20, 24 (19)	4, 6, 6, 10 (7)
% of SOC instances, each type of SOC	ES	44	8	19	23
	LBS	32	34	40	54
	O	17	3	7	0
	C	7	55	34	23
	Total	100	100	100	100

*Note:* C = Compensation; ES = Elective Selection; LBS = Loss-Based Selection; O = Optimization; PF = physical function; SOC = Selection, Optimization, and Compensation; WB = well-being.

Different levels of physical function were associated with different types of SOC talk. Group 1, which had the highest physical function, spoke more than other groups about ES and Optimization. Group 2 had the lowest physical function and used Compensation the most. Group 3 had slightly higher physical function and used a broad mix of SOC strategies. Group 4 engaged in little SOC.

In the remainder of this section, we first provide an overview of interviewees’ SOC talk. We then draw on content and narrative analyses to compare the four groups, discussing how their differing patterns of SOC talk might be interpreted in terms of successful aging.

### Elective Selection

Members of Group 1 engaged in relatively high amounts of ES, a process that appeared to be related to their high level of physical functioning. This high level of ES included undertaking new, physically demanding activities. Brenda, for example, had recently adopted a puppy and had begun volunteering in the local hospital garden. Colin had taken up hillwalking (see Table 3), and the contrast between his own activities and those of Catherine (Group 3), who had low physical function, is potentially instructive. Whereas Catherine was forced to give up hillwalking in favor of a less strenuous variant of that activity—walking “to the hills rather than up them” (LBS)—Colin was able to add hillwalking to an already crowded list of physically demanding activities (ES).

### Loss-Based Selection

LBS was an important strategy for all groups, and interviewees spoke of two primary approaches to this strategy: using LBS to continue engaging in cherished activities but in less resource intensive forms and using LBS to choose from among a range of goal-relevant activities.

LBS also appeared to play an important role in identity maintenance. A number of men in the qualitative subsample were lifelong handymen and spoke of using LBS to continue doing routine domestic repairs and improvements, despite physical decline. As Neil (Group 1) said:

I don’t do ladder work now. I think there was a year when I felt my wrists weren’t strong enough to deal with the ladder, you know, and I just said, ‘Right, that’s it.’ I stopped.

However, Neil went on to emphasize that he continued to do all the “normal maintenance” in his home and did not have to seek help for anything other than ladder work. Owen (Group 3) suffered from worse physical function than Neil but provided a similar narrative, saying that whereas he no longer climbed ladders, he could still “do the woodwork” and perform other domestic improvements. In their late 70s, these and other men were now forced to forego the most physically demanding aspects of an activity that had long been important to their identity. However, they were able to use LBS to continue engaging in that activity and to continue seeing themselves as men who were handy around the house. LBS thus offered these men a means for maintaining identity in the face of physical decline. Women’s narratives of household-related LBS typically had a slightly different structure. Whereas men were more likely to provide narratives in which they continued engaging in important activities but in less resource intensive forms, women were more likely to describe cutting back on one resource intensive activity, particularly housework, in order to devote their reduced physical resources to other, more favored activities, especially gardening.

### Optimization

Optimization was the least commonly cited strategy. This finding conflicts with that of Gignac and colleagues (2002), who found that Optimization was less prevalent than Compensation but more common than Selection. However, our finding matches that of Rozario and colleagues (2011), who found limited use of Optimization and concluded that this strategy requires a greater level of physical resource than is available to many older people. Freund (2008) has suggested that, as resources decline, it becomes increasingly difficult or costly to invest in efforts to maximize functioning. This would indicate that Optimization is positively associated with higher physical function, a conclusion matched by our own findings. Group 1, which had the highest physical function, made the greatest use of Optimization, often to improve their already good health. For example, Neil said that he used a Wii because, “I think that helps your timing, your coordination.” Colin had recently begun “making myself go to the gymnasium every day” in an effort to improve his physical and mental fitness. Working out was “very painful,” he said, but necessary if he wanted to achieve his health-related goals. In this example, Colin goes to the gym not because it is an activity he enjoys, but because it improves his ability to engage in resource intensive activities such as hillwalking. As such, he is able to establish a virtuous cycle in which high physical function allows him to pursue even greater physical health. The other three groups made little to no use of Optimization.

### Compensation

Compensation was particularly important for individuals with low physical function. Compensation devices typically took two forms: tools such as walking sticks, and adaptations to one’s home. Several interviewees said they had made such adaptations in order to avoid or at least delay being forced out of the family home by physical decline. For example, Owen (Group 3), who had low physical function, had installed a set of handrails on the stairs and said they had

stood us in good stead.… It’s nice, when you happen to meet your wife coming down and she’s holding one of them [and you’re holding] another one on the other side.

Attitudes to Compensation strategies were nuanced and reflected a desire to remain as autonomous as possible. Mary (Group 3) said

After the first [hip] operation I wasn’t allowed to bend, so I’d sweep the kitchen floor and then I’d have to get [HUSBAND] to come in and put it into a dustpan. That really irritated me no end. He wasn’t irritated but, you know, I was. And I got one of the long-handled dustpans and brushes…and it’s been fabulous.

Though help from another is a form of Compensation, accepting such help had negative impacts on Mary’s well-being, perhaps because it highlighted her diminished autonomy (Rozario et al., 2011). Maintenance of autonomy and physical function were important goals for interviewees, including those with the poorest physical health. Eleanor (Group 2), whose arthritis was so severe it was “like walking on glass,” said that even though she struggled to get around without a mobility scooter, she forced herself to use Compensation devices such as a walking stick instead, to maintain her strength and mobility. However, she did allow herself to use a scooter when on vacation: “You’re away from home so you can do that kind of thing.”

### Comparing the Four Groups

Group 1 had high well-being and physical function and engaged in medial amounts of SOC talk. As might be expected given their high physical function, this group engaged in little Compensation but relatively high levels of ES and Optimization. From a narrative perspective, this group used SOC to add new chapters to their life stories by adopting new activities and pursuing new goals. Group 1 also had high expectations regarding future chapters. For example, James said that he had recently told his young granddaughter that he planned “to be able to dance at your wedding”—an event that was unlikely to occur before he was 100 years. A high user of SOC, James felt confident that he could sustain a healthy and dynamic Third Age (Laslett, 1989) for decades to come.

Group 2 had low well-being and physical function. This group engaged in almost as much SOC talk as Group 1, and approximately twice as much as the other low well-being group (Group 4). Group 2 showed greater reliance on Compensation than did groups with higher physical function. Despite poor physical health, including severe chronic pain, members of Group 2 used SOC, especially Compensation and LBS, to continue pursuing meaningful goals, albeit in reduced form. Eleanor, for example, was a lifelong dog lover and gardener but could no longer engage in these activities, so she had “downsized” to a cat and had shifted from being a “proper gardener” to a “potted gardener” (LBS). From a narrative perspective, Group 2 used SOC to forestall narrative foreclosure (Freeman, 2000) and fight “against the dying of the light” (Thomas, 1952). We suggest that this group could be perceived as “struggling,” with that term understood in both its primary senses, that is, (a) suffering but (b) continuing to fight on.

Group 3 was “off-diagonal,” with high well-being despite low physical function. This group engaged in more SOC talk than the other three groups and, like Group 1, featured a varied mix of different types of SOC talk. However, whereas Group 1’s SOC talk was weighted toward ES, Group 3 featured high levels of LBS and Compensation.

Like Group 2, who also suffered from low physical function, Group 3 spoke of pursuing fewer goals than before. Unlike the former group, however, Group 3 still strived to pursue key goals in a non-diminished form. For example, Mary appeared to see gardening as central to her identity, and thus prioritized its role in her life. She used Compensation and LBS to continue maintaining a large garden, and refused to downsize to what she referred to as “a geriatric garden,” despite severe mobility problems and several garden-induced falls. Another member of Group 3, Catherine, said that even though she suffered worse health than many of her peers, she remained more active, thanks to skilful use of Compensation and LBS. Thus, despite poor physical function, members of Group 2 continued to pursue valued goals and strived to keep key chapters of their life stories developing.

Group 4 was off-diagonal in that it had low well-being despite relatively high physical function. Quantitatively, this group engaged in much less SOC talk than their peers: roughly half as much as Groups 1 and 2, and one-third as much as Group 3. Narratively, a key difference between this and the other groups was the lack of adaptive resolutions in Group 4’s discussions of physical decline. All groups provided narratives of physical decline and its impact on activities. These narratives typically took the form of:

(1) Because of X (some form of physical decline),
(2) I had to give up Y (a cherished activity).

Groups 1–3 typically transformed these stories into narratives of adaptive resolution, that is, narratives in which LBS or Compensation was used to adapt to decline and continue pursuing valued goals. These adaptive resolutions typically took the following forms:

(3) So I started doing a less demanding version of Y (e.g., walking to the hills rather than up them) OR
(4) But that left more time and energy to focus on Z (an activity deemed more important or manageable than Y, e.g., gardening rather than housework).

For example, Mary (Group 3) provides an example in which the loss of one physical activity is made up for by the ability to engage in a more preferred activity:

I can’t see me being able to play golf again…I’ve got a very stiff neck and just trying to look to see where my ball’s going [is] uncomfortable. But…I’ve got plenty to keep me going anyway just here in the garden.

For Group 4, however, narratives of activity loss typically ended without adaptive resolutions—for example, Ian said, “I used to do a lot of shooting…and I don’t shoot now.” The narrative ends there, without moving onto an activity he has replaced shooting with. Rather than pursue a smaller number of goals, or less demanding ones, Group 4 seemed more willing to allow chapters of their life story to close. At least in the physical domain, they appeared to engage in relatively little SOC to adapt to the losses associated with aging.

In summary, Group 1 took advantage of their good physical function to engage in high levels of ES, pursue new goals alongside old ones, and add new chapters to their life story. Group 2 primarily used Compensation and LBS to adapt to low physical function, maintain the pursuit of valued goals (albeit in reduced form), and to slow the closing of valued chapters in their life story, that is, to avoid narrative foreclosure. Group 3 maintained high well-being despite poor physical function and engaged in extensive SOC to continue pursuing important goals and to keep key life chapters developing. Group 4 had low well-being despite relatively good physical function and made very little use of SOC.

## Discussion

We found support for our hypothesis that older people who maintained high well-being despite low physical function would engage in more SOC talk than their peers, and those who reported lower well-being despite relatively high physical function would engage in less. Group 3, who had high well-being despite low physical function, engaged in almost three times as much SOC talk as Group 4, who had higher physical function but low well-being. The two “on-diagonal” groups both engaged in medial levels of SOC talk. This finding suggests that SOC may play a role in mediating the relationship between physical function and well-being. Ziegelmann and Lippke (2007) reached a similar conclusion in a quantitative study, finding that SOC supported higher levels of physical activity and well-being.

Our analysis has methodological and theoretical implications for the study of SOC specifically and successful aging more generally. Methodologically, we add to the limited body of qualitative research on SOC. Our qualitative approach to everyday SOC behaviors complements and adds to quantitative research on general SOC propensities by providing insights into how, when, why, and in what ways older people incorporate SOC into their daily lives to maximize well-being. Even when older people were not explicitly asked about SOC, they frequently talked about it in relation to everyday activities, indicating that it is possible to study these processes in a general qualitative data source (see also Rozario et al., 2011).

Theoretically, our analysis brings together outcome- and process-focused approaches to successful aging. Group 1 appeared to be aging successfully both in terms of outcomes and processes: This group had high well-being and physical function and used SOC to pursue new goals and add new chapters to their life story. We would argue that Group 3 should also be seen as aging successfully: Despite low physical function, this group had high well-being and appeared to make extensive use of SOC to remain active, continue pursuing valued goals, and keep key chapters of their life story developing. This successful use of SOC brings to mind Young and colleagues (2009, pp. 88–89), who conceptualize successful aging as, “a state wherein an individual is able to invoke adaptive psychological and/or social mechanisms to compensate for physiological limitations to achieve a sense of well-being, high self-assessed quality of life, and a sense of personal fulfilment.” From a policy and program standpoint, Group 3’s use of SOC may be particularly instructive. Whereas one important policy question is how to influence the life course so as to maximize outcomes in old age—that is, how to increase the proportion of older people with high well-being and high physical function—another key question is how to maximize well-being in old age despite the physical decline that eventually reshapes most individuals’ lives. Although it is not feasible for everyone to maintain high physical function into their late 70s and beyond, everyone can be encouraged and supported to continue engaging in valued activities and pursuing goals as they age and provided with practical strategies for doing so.

In this light, it may be instructive to compare Group 2, which had poor scores on both outcome measures, to Group 4, which had low well-being despite relatively high physical function. Outcome-only definitions of successful aging would suggest that Group 4 was aging more successfully than Group 2 because of the former group’s higher physical function. However, Group 4 showed very limited use of SOC and thus appeared more prone to narrative foreclosure, at least in the physical domain. If definitions of successful aging are to include outcome and process focuses, as perhaps they should (especially when considering the older old, for whom high physical function is unlikely), Group 4 may be aging less successfully than Group 2, despite better physical function.

Our study has a number of limitations, including the size of the qualitative sample and the cross-sectional nature of the data drawn on for this article. Future research should draw on larger samples, particularly for the off-diagonal groups. On this project, coding of SOC talk was done by researchers who were aware of the interviewees’ physical function and well-being scores, introducing potential bias into the coding process. However, the SOC talk quantification process may also help to improve the rigor of the results, by ensuring that qualitative claims about amounts and types of SOC talk were clearly grounded in numerical tallies, thus reducing the likelihood of confirmation bias.

## Conclusion

Atkinson (2009) has criticized the tendency of some narrative research to valorize narratives for their own sake. Quantifying SOC talk and drawing on quantitative outcome data helped us to avoid this. While putting older people’s voices at the center of our study, we analyzed SOC talk in terms of its association with physical function and well-being. Based on this, we suggest that, whereas there are a range of valid approaches to the conceptualization of successful aging, a full definition that will be applicable into the ninth decade of life and beyond should include subjective and objective components (Pruchno & Wilson-Genderson, 2015) and process- and outcome-focused approaches (Kahana et al., 2012). Our analysis brings together these approaches to successful aging and provides evidence of the ways that SOC may help older people to continue developing their life stories, avoid narrative foreclosure, and maximize well-being despite physical loss.

Whereas some scholars (e.g., Katz & Calasanti, 2015; Martinson & Berridge, 2015) have suggested that the concept of successful aging should be abandoned in favor of other approaches, we argue that this “simple, intuitive little phrase” has a “visceral, hard-to-put-your-finger-on appeal” (Glass, 2003, p. 382). We therefore concur with Rowe and Kahn’s (2015) call for conceptual expansion rather than abandonment. Part of the appeal of successful aging as a concept, we suggest, is its narrative quality (Carpentieri et al., 2016). There is an inherently narrative aspect to the challenge of aging well: one must strive to keep one’s life story developing despite physical decline and other losses. In this article, we have provided evidence, from the perspectives of older people themselves, of the ways in which SOC may play a role in that process.

## Funding

The authors were supported by a Research Council UK Life Long Health and Wellbeing Programme grant (MRC G1001401/1). I. D. Deary and C. E. Brett are supported by the Centre for Cognitive Ageing and Cognitive Epidemiology, part of the cross-council Life Long Health and Wellbeing Initiative (MR/K026992/1). Funding from the Biotechnology and Biological Sciences Research Council (BBSRC) and Medical Research Council (MRC) is gratefully acknowledged, as is the valuable contribution of Roona Simpson.

## Conflict of Interest

The authors declare no conflicts of interest.
